# Prognostic value of C-reactive protein in patients with melanoma: a meta-analysis

**DOI:** 10.1080/07853890.2025.2521446

**Published:** 2025-06-20

**Authors:** Xuejia Xing, Zhixiang Fang

**Affiliations:** Clinical Laboratory, Changxing People’s Hospital, Huzhou, Zhejiang, China

**Keywords:** C-reactive protein, melanoma, meta-analysis, prognosis, biomarker

## Abstract

**Background:**

C-reactive protein (CRP) has been widely investigated for its prognostic value for melanoma, however, findings remained different. Consequently, this meta-analysis was performed for identifying its precise role in forecasting melanoma prognosis.

**Methods:**

We searched PubMed, Web of Science, Embase and Cochrane Library till 25 January 2025, and computed combined hazard ratios (HRs) and 95% confidence intervals (CIs) for estimating CRP’s effect on forecasting overall survival (OS) and progression-free survival (PFS) of melanoma.

**Results:**

Nineteen studies consisting of 4634 cases were enrolled into the present work. As suggested by our data, higher CRP showed remarkable relation to dismal OS (HR = 2.26, 95% CI = 1.89–2.71, *p* < 0.001) and inferior PFS (HR = 1.84, 95% CI = 1.13–2.98, *p* = 0.014) of melanoma patients. Subgroup analyses indicated that CRP had consistent prognostic significance of OS among various subgroups (all *p* < 0.05). The findings were verified to be reliable by publication bias and sensitivity analyses.

**Conclusion:**

High CRP remarkably predicts dismal OS and PFS of melanoma cases, which is the promising biomarker used to forecast melanoma prognosis in clinical practice.

## KEY MESSAGES

1. To our knowledge, the present work provides the initial meta-analysis on CRP for its value in forecasting melanoma prognosis.
2. Higher CRP showed remarkable relation to dismal OS and inferior PFS of melanoma patients.
3. CRP is the promising biomarker used to forecast melanoma prognosis in clinical practice.

## Introduction

Melanoma remains the leading cause of death from skin cancer, with its occurrence consistently increasing [1]. According to GLOBOCAN, 331,647 newly diagnosed together with 58,645 death cases due to this melanoma were reported in 2022 globally [2]. Melanoma arises from the malignant alteration and uncontrolled multiplication of melanocytes, making it a highly aggressive disease [3]. While 65 is the average age for diagnosis, individuals of any age can develop melanoma [4]. Previously, patients diagnosed with stage IV melanoma by the American Joint Committee on Cancer had a very poor prognosis when untreated, and the median 1-year and 5-year survival rates were <10% [5]. The treatment of melanoma has greatly progressed with the introduction of groundbreaking immunotherapy, including anti-PD-(L)1 and anti-CTLA-4 antibodies [6]. Among melanoma patients, 50% will carry a BRAF mutation, and BRAF- and MEK-targeted therapies are effective treatment options for these cases [7]. The early-stage diagnosis of almost 90% of melanomas emphasizes the unpredictable nature of tumour behaviour and the limitations of existing staging systems in accurately assessing and forecasting melanoma patient outcomes [8]. Consequently, identifying reliable biomarkers for prognosis forecasting is pivotal for improving the survival outcomes of patients with melanoma.

C-reactive protein (CRP), predominantly produced by the liver, is a typical acute-phase protein and one of the most routinely measured indicators of systemic inflammation [9]. CRP is commonly known among physicians as a marker for inflammation. CRP is previously suggested to forecast prognosis of diverse cancers, including breast cancer [10], pancreatic cancer [11], non-small cell lung cancer (NSCLC) [12], diffuse large B-cell lymphoma [13] and oesophageal cancer [14]. CRP is extensively suggested for its value in forecasting melanoma prognosis, but findings remained inconsistent [15–33]. For example, in some studies, higher CRP was markedly related to dismal prognosis of melanoma [19–22,24–26,31,32]. A recent study including two cohorts showed that initial CRP kinetics might suggest a reaction to immune checkpoint inhibition (ICI) with enhanced survival outcomes in melanoma [34]. Outcomes are significantly better in cases of CRP flare and response than in CRP non-responders [34]. However, other investigators discovered that there was no significant correlation between CRP and survival outcomes of patients with melanoma [23,27]. Therefore, we performed this meta-analysis to identify the precise prognostic role of CRP in melanoma.

## Materials and methods

### Study guideline

This study was conducted according to the Preferred Reporting Items for Systematic Reviews and Meta-Analyses (PRISMA) guideline [35].

### Literature search

We searched PubMed, Web of Science, Embase and Cochrane Library. The search timeline was from inception to 25 January 2025. The search strategies were: (CRP OR C-reactive protein) AND (melanoma OR malignant melanoma). Only English publications were enrolled. Additionally, references and related reviews were manually retrieved to find potentially relevant studies.

### Inclusion and exclusion criteria

Studies below were included: (1) cases with pathological diagnosis of any type of melanoma; (2) studies exploring the correlation of CRP with survival in melanoma cases; (3) CRP levels were tested pretreatment because it was the baseline and was not affected by treatment; (4) those reporting hazard ratios (HRs) as well as 95% confidence intervals (CIs); (5) those with the definite threshold CRP; and (6) English studies. Studies below were eliminated: (1) case reports, reviews, meeting abstracts; comments and letters; (2) those with duplicate patients; and (3) animal studies.

### Data extraction and quality assessment

Two researchers (X.X. and Z.F.) extracted data from qualified articles and all disagreements were resolved by discussion. The following information were extracted: author, year, country, sample size, gender, age, study design, study period, study centre, tumour type, TNM stage, treatment, cut-off value, survival endpoints, follow-up, survival analysis types and HRs with 95% CIs. Overall survival (OS) and progression-free survival (PFS) were our primary and secondary survival outcomes separately. Newcastle-Ottawa Scale (NOS) was applied in evaluating study quality [36], and scores were 0–9, with NOS ≥6 suggesting high quality.

### Statistical analysis

We predicted CRP for its value in forecasting OS and PFS of melanoma through computing combined HRs and 95% CIs. Among-study heterogeneities were assessed through Cochran’s Q chi-square test and I^2^ statistics. If the I^2^ value was over 50% or the Q-test yielded a *p*-value under 0.10, showing obvious heterogeneity, the random-effects model would be employed. Otherwise, we applied the fixed-effects model. Subgroup analyses were conducted for investigating CRP’s effect on forecasting prognosis of different patients’ populations of melanoma. We also implemented sensitivity analysis for evaluating whether our findings were stable through eliminating articles one by one from the pooled analyses. Funnel plots, Begg’s and Egger’s tests were adopted for evaluating publication bias. *p* < 0.05 stood for statistical difference. Stata 12.0 software (Stata Corp, College Station, TX, USA) was employed for statistical analysis.

## Results

### Process of literature search

**Figure 1.**
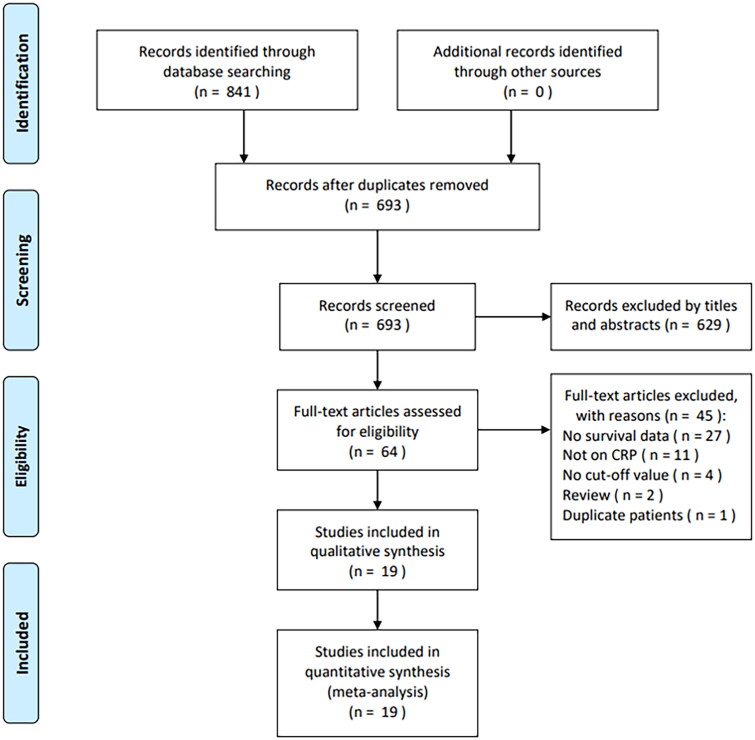
PRISMA diagram of the study selection.

Through primary search, altogether 841 studies were obtained, among which, 693 were retained when duplicates were eliminated (Figure 1). Upon title- and abstract-reviewing, we excluded 629 records due to irrelevance and animal studies. Later, we tested 64 articles through reading full-texts and discarded 45 of them due to the following reasons: no survival data provided (*n* = 27), not on CRP (*n* = 11), no cut-off value (*n* = 4), review (*n* = 2) and duplicate patients recruitment (*n* = 1). Finally, 19 studies consisting of 4634 patients [15–33] were included in this meta-analysis (Figure 1).

### Characteristics of included studies

The publication year of all enrolled articles was from 1996 to 2025 and they were all published in English [15–33] (Table 1). Five studies were conducted in Germany [23,24,28,29,32], two studies were performed in Italy [16,19], two studies were carried out in Norway [26,33], two in USA [20,27] and one each in France [15], Austria [17], Belgium [18], Czech [21], Japan [22], China [25], Switzerland [30] and UK [31], respectively. Sample sizes were 30–1144 (median, 101). There were eight prospective studies [15,16,20,21,26,27,31,33] and 11 retrospective studies [17–19,22–25,28–30,32] (Table 1). Ten studies were single centre studies [17–20,22,23,25,28,30,32] and nine were multicentre trials [15,16,21,24,26,27,29,31,33]. Ten studies enrolled patients with stage IV [15–18,24,26–29,33], six studies recruited patients with stage III-IV [19–22,30,31] and one each included patients with stage I-IV [23], I-III [25] and III [32], respectively. Regarding treatment methods, 10 studies used immune checkpoint inhibitors (ICIs) immunotherapy [18,19,21,22,24,26,27,30,31,33], five studies applied surgical resection [17,25,28,29,32], two studies used multimodality therapy [20,23] and one each used chemotherapy [16] and interleukin-2 (IL-2) regimen [15], respectively. The threshold CRP was 3–20 (mg/L) (median, 10). All 19 articles reported CRP’s effect on forecasting OS [15–33], while five presented the relation of CRP with PFS [17,23,30–32] in melanoma. Sixteen articles obtained the HRs and 95% CIs upon univariate regression [15–19,21–27,29–32] and three studies used multivariate analysis [20,28,33]. For all those qualified articles, their NOS scores were 7-9, demonstrating their high quality [15–33] (Table 1).

**Table 1.** Baseline characteristics of studies included in this meta-analysis.

Study	Year	Country	Sample size	Gender (M/F)	Age (year) Median (range)	Study design	Study period	Study centre	Tumour type	TNM stage	Treatment	Cut-off value (mg/L)	Survival outcome	Follow-up (month) Median (range)	Survival analysis	NOS score
Tartour	1996	France	101	50/51	50 (20–66)	Prospective	NR	Multicentre	Mixed	IV	IL-2 regimen	10	OS	1–48	Univariate	8
Guida	2003	Italy	176	105/71	59 (26–76)	Prospective	1997–1999	Multicentre	Mixed	IV	Chemotherapy	10	OS	16 (1–21)	Univariate	8
Schicher	2013	Austria	49	25/24	57 (23–87)	Retrospective	2000–2010	Single centre	Uveal	IV	Surgery	5	OS, PFS	1–120	Univariate	7
Wilgenhof	2013	Belgium	50	28/22	50 (31–80)	Retrospective	2010–2011	Single centre	Non-uveal	IV	Immunotherapy	5	OS	1–26	Univariate	7
Simeone	2014	Italy	95	54/41	58 (17–84)	Retrospective	2010–2012	Single centre	Mixed	III-IV	Immunotherapy	8	OS	24 (1–36)	Univariate	7
Fang	2015	USA	1144	652/492	55	Prospective	1998–2009	Single centre	Cutaneous	III-IV	Mixed	10	OS	74 (1–168)	Multivariate	9
Krajsová	2015	Czech	196	112/84	61 (22–86)	Prospective	2010–2012	Multicentre	Mixed	III-IV	Immunotherapy	10	OS	39 (27–47)	Univariate	9
Nakamura	2016	Japan	98	52/46	66 (17–93)	Retrospective	2014–2016	Single centre	Mixed	III-IV	Immunotherapy	3	OS	1–25	Univariate	7
Desch	2017	Germany	533	314/219	64 (21–94)	Retrospective	2010–2015	Single centre	Mixed	I-IV	Mixed	5	OS, PFS	1–60	Univariate	8
Heppt	2017	Germany	54	29/25	<60: 15 ≥ 60: 39	Retrospective	Jul-Oct 2016	Multicentre	Uveal	IV	Immunotherapy	10	OS	1–25	Univariate	7
Wang	2017	China	232	123/109	54 (18–84)	Retrospective	2000–2010	Single centre	Acral	I-III	Surgery	5	OS	1–144	Univariate	8
Nyakas	2019	Norway	56	33/23	60 (27–83)	Prospective	2014–2015	Multicentre	Mixed	IV	Immunotherapy	20	OS	1–40	Univariate	9
Laino	2020	USA	945	526/419	55 (23–86)	Prospective	2013–2014	Multicentre	Mixed	IV	Immunotherapy	10	OS	1–54	Univariate	9
Ludwig	2021	Germany	54	24/30	61 (26–81)	Retrospective	2014–2015	Single centre	Uveal	IV	Surgery	10	OS	15.8 (1–35)	Multivariate	7
Schneider	2021	Germany	30	19/11	62 (34–84)	Retrospective	2013–2019	Multicentre	Mixed	IV	Surgery	10	OS	1–96	Univariate	8
Boerlin	2022	Switzerland	79	51/28	62 (24–88)	Retrospective	2015–2021	Single centre	Cutaneous	III-IV	Immunotherapy	10	OS, PFS	1–60	Univariate	7
Larkin	2023	UK	453	258/195	56 (19–83)	Prospective	Mar-Nov 2015	Multicentre	Mixed	III-IV	Immunotherapy	10	OS, PFS	18–69	Univariate	9
Schildbach	2023	Germany	138	71/67	59 (45–72)	Retrospective	2011–2020	Single centre	Cutaneous	III	Surgery	3	OS, PFS	53.4 (1–120)	Univariate	8
Lereim	2025	Norway	151	96/55	63 (27–84)	Prospective	2014–2015	Multicentre	Mixed	IV	Immunotherapy	10	OS	1–72	Multivariate	9

NR, not reported; OS, overall survival; PFS, progression-free survival; NOS, Newcastle-Ottawa Scale.

Treatment, mixed refers to multimodality therapy including surgery, immunotherapy, and targeted therapy.

### CRP and OS

**Figure 2.**
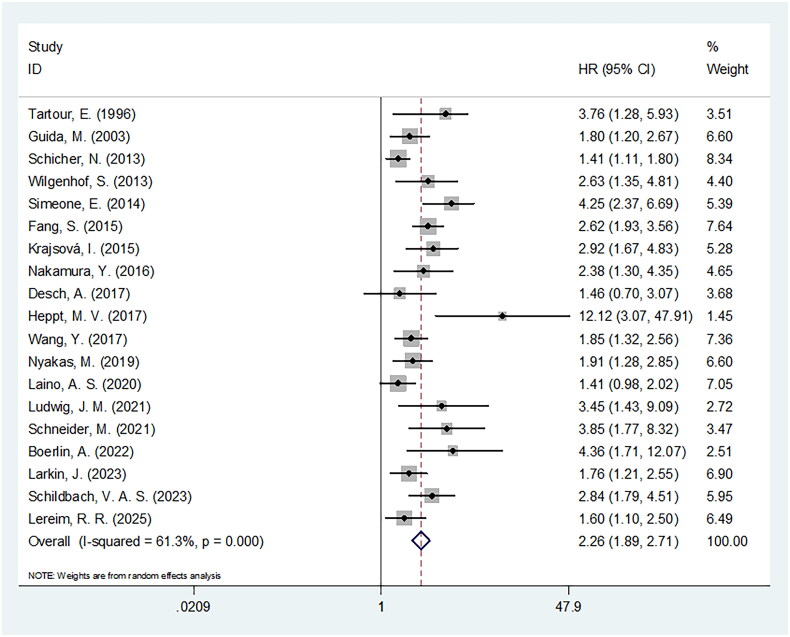
The forest plots for the association between CRP and OS in patients with melanoma.

All 19 studies with 4634 patients [15–33] reported the prognostic role of CRP for OS in melanoma. A random-effects model was applied because of significant heterogeneity (I2 = 61.3%, *p* < 0.001; Table 2). The pooled results were: HR = 2.26, 95% CI = 1.89–2.71, *p* < 0.001, suggesting the remarkable relation of elevated CRP with dismal OS of melanoma (Table 2 and Figure 2). As demonstrated by subgroup analyses, CRP exerted prominent value for forecasting OS, which was independent of country, sample size, study design, study centre, tumour type, TNM stage, treatment, threshold and survival analysis types (all *p* < 0.05; Table 2).

**Table 2.** Subgroup analysis of prognostic value of CRP for OS in patients with melanoma.

Subgroups	No. of studies	No. of patients	Effects model	HR (95% CI)	*p*	Heterogeneity	
						I2 (%)	Ph
**Total**	19	4634	Random	2.26 (1.89–2.71)	<0.001	61.3	<0.001
**Country**							
Western	17	4304	Random	2.32 (1.89–2.84)	<0.001	65.1	<0.001
Asian	2	330	Fixed	1.96 (1.47–2.62)	<0.001	0	0.473
**Sample size**							
<100	9	565	Random	2.87 (1.96–4.20)	<0.001	73.5	<0.001
≥100	10	4069	Fixed	2.00 (1.76–2.28)	<0.001	44.3	0.063
**Study design**							
Prospective	8	3222	Fixed	1.98 (1.71–2.28)	<0.001	46.4	0.071
Retrospective	11	1412	Random	2.65 (1.94–3.61)	<0.001	69.8	<0.001
**Study centre**							
Single centre	10	2472	Random	2.39 (1.84–3.10)	<0.001	66.1	0.002
Multicentre	9	2162	Random	2.14 (1.65–2.78)	<0.001	58.5	0.014
**Tumour type**							
Mixed	11	2834	Random	2.14 (1.72–2.68)	<0.001	54.5	0.015
Cutaneous	3	1361	Fixed	2.77 (2.16–3.55)	<0.001	0	0.617
Uveal	3	157	Random	3.32 (1.05–10.51)	0.041	83.4	0.002
Acral	1	232	–	1.85 (1.32–2.56)	<0.001	–	–
Non-Uveal	1	50	–	2.63 (1.35–4.81)	<0.001	–	–
**TNM stage**							
I-III/I-IV/III	3	903	Fixed	2.06 (1.46–2.89)	<0.001	35.5	0.212
III-IV	6	2065	Fixed	2.68 (2.05–3.51)	<0.001	44.4	0.109
IV	10	1666	Random	1.76 (1.53–2.02)	<0.001	62.4	0.004
**Treatment**							
Immunotherapy	10	2177	Random	2.36 (1.79–3.12)	<0.001	64.2	0.003
Surgery	5	503	Random	2.22 (1.53–3.21)	<0.001	69.9	0.010
Mixed	2	1677	Random	2.16 (1.26–3.70)	0.005	51.3	0.152
Chemotherapy/IL-2	2	277	Random	2.41 (1.19–4.89)	0.015	64.1	0.095
**Cut-off value (mg/L)**							
10	11	3383	Random	2.39 (1.85–3.09)	<0.001	60.6	0.005
≠10	8	1251	Random	2.15 (1.64–2.80)	<0.001	65.1	0.005
**Survival analysis**							
Univariate	16	3285	Random	2.28 (1.86–2.80)	<0.001	63.4	<0.001
Multivariate	3	1349	Random	2.26 (1.50–3.39)	<0.001	54.7	0.110

### CRP and PFS

**Figure 3.**
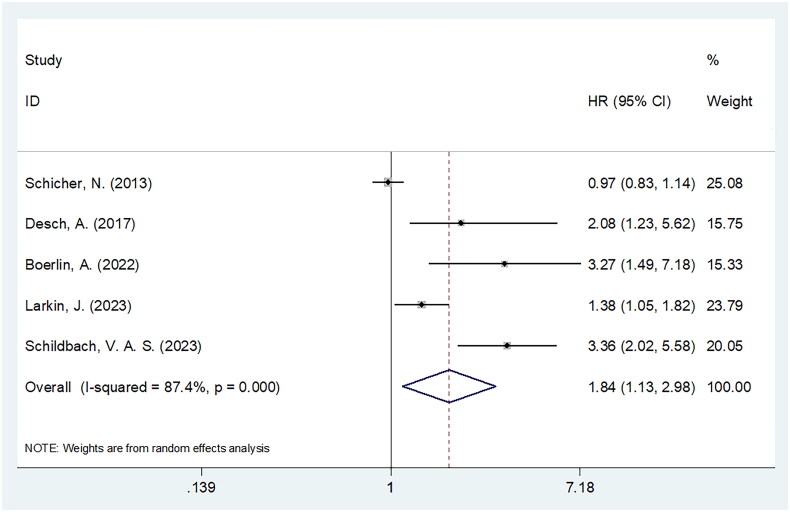
The forest plots for the association between CRP and PFS in patients with melanoma.

Five studies comprising 1252 patients [17,23,30–32] presented the correlation between CRP and PFS in melanoma. The combined data showed the obvious relation of high CRP with dismal PFS in melanoma (HR = 1.84, 95% CI = 1.13–2.98, *p* = 0.014) (Table 3 and Figure 3). As revealed by subgroup analyses, CRP still markedly forecast poor PFS of subgroups below: sample size ≥100 (*p* = 0.020), prospective study design (*p* = 0.022), single centre study (*p* = 0.022), mixed tumour type (*p* = 0.005), cutaneous melanoma (*p* < 0.001) and TNM I-III/I-IV/III stages (*p* < 0.001) (Table 3).

**Table 3.** Subgroup analysis of prognostic value of CRP for PFS in patients with melanoma.

Subgroups	No. of studies	No. of patients	Effects model	HR (95% CI)	*p*	Heterogeneity	
						I2 (%)	Ph
**Total**	5	1252	Random	1.84 (1.13–2.98)	0.014	87.4	<0.001
**Sample size**							
<100	2	128	Random	1.67 (0.51–5.46)	0.395	88.7	0.003
≥100	3	1124	Random	2.07 (1.12–3.82)	0.020	78.5	0.009
**Study design**							
Prospective	1	453	–	1.38 (1.05–1.82)	0.022	–	–
Retrospective	4	799	Random	2.09 (0.95–4.61)	0.066	90.2	<0.001
**Study centre**							
Single centre	1	453	–	1.38 (1.05–1.82)	0.022	–	–
Multicentre	4	799	Random	2.09 (0.95–4.61)	0.066	90.2	<0.001
**Tumour type**							
Mixed	2	986	Fixed	1.45 (1.12–1.87)	0.005	0	0.320
Cutaneous	2	217	Fixed	3.33 (2.17–5.10)	<0.001	0	0.957
Uveal	1	49	–	0.97 (0.83–1.14)	0.707	–	–
TNM stage							
I-III/I-IV/III	2	671	Fixed	2.89 (1.90–4.42)	<0.001	4.8	0.305
III-IV	2	532	Random	1.96 (0.85–4.49)	0.113	75.7	0.042
IV	1	49	–	0.97 (0.83–1.14)	0.707	–	–
**Treatment**							
Immunotherapy	2	532	Random	1.96 (0.85–4.49)	0.113	75.7	0.042
Surgery	2	187	Random	1.76 (0.52–5.93)	0.362	95.2	<0.001
Mixed	1	533	–	2.08 (0.97–4.45)	0.059	–	–
**Cut-off value (mg/L)**							
10	2	532	Random	1.96 (0.85–4.49)	0.113	75.7	0.042
≠10	3	720	Random	1.84 (0.75–4.50)	0.182	91.5	<0.001

### Sensitivity analysis

**Figure 4.**
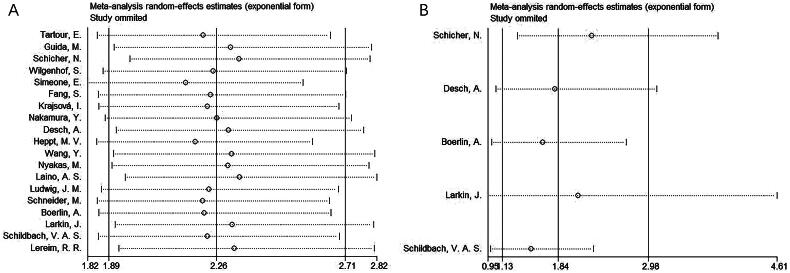
Sensitivity analysis. (a) OS and (b) PFS.

We applied sensitivity analysis to OS and PFS data for assessing how every cohort study contributed to overall HR through eliminating them in sequence. According to our findings, overall HRs of OS and PFS were stable, even when each cohort study was sequentially omitted (Figure 4).

### Publication bias

**Figure 5.**
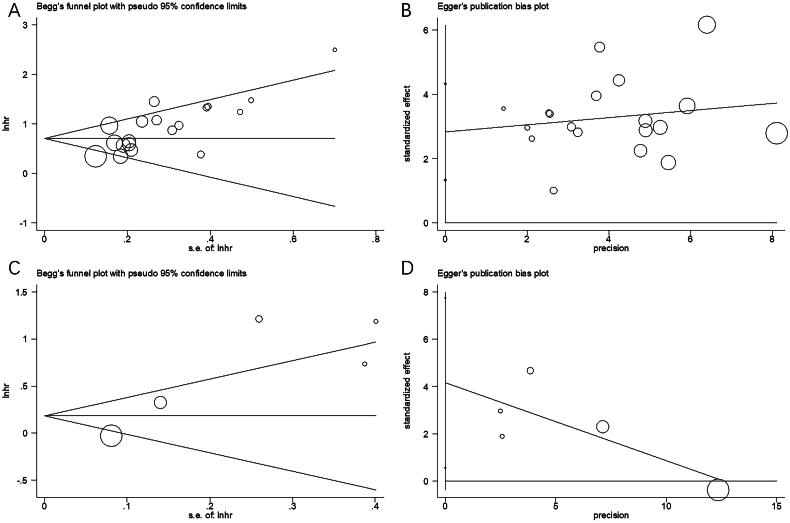
Publication bias by Begg’s test and Egger’s test. (a) Begg’s test for OS, *p* = 0.214; (b) Egger’s test for OS, *p* = 0.201; (c) Begg’s test for PFS, *p* = 0.221; and (d) Egger’s test for PFS, *p* = 0.135.

Funnel plots, Begg’s and Egge’s tests were performed for assessing possible publication bias. From Figure 5, funnel plots revealed symmetry, indicating no obvious publication bias related to OS (*p* = 0.214/0.201 through Begg’s/Egger’s tests) or PFS *p* = 0.221/0.135 through Begg’s/Egger’s tests) in this meta-analysis (Figure 5).

## Discussion

CRP plays a controversial role in the prognosis of melanoma. This study aggregated data from 19 studies with 4634 patients to investigate its precise significance for forecasting melanoma prognosis. Notably, in this meta-analysis, CRP levels were tested pretreatment because it was the baseline and was not affected by treatment. As revealed by the findings, elevated CRP apparently forecast poor OS and inferior PFS of melanoma. Subgroup analyses indicated consistent value of CRP in forecasting OS in various subgroups. Besides, these findings were verified to be reliable through publication bias and sensitivity analyses. Therefore, CRP could be employed as a promising prognostic marker to predict short- and long-time survival outcomes of melanoma. The present work provides the initial meta-analysis on CRP for its value in forecasting melanoma prognosis.

Increased levels of CRP in the inflammatory and tumour microenvironment (TME) facilitate the development of various cancers, like breast, liver, renal and pancreatic cancer, by engaging with different inflammatory molecules [37]. Exact mechanisms related to the exact significance of CRP in forecasting melanoma prognosis remain to be further clarified, they are interpreted as follows. Firstly, research has shown that CRP is linked to the development of an inflammatory tumour microenvironment and the activation of specific signalling pathways involved in tumour growth, spread and blood vessel formation [38]. Interleukin-6 (IL-6), a key cytokine in the tumour microenvironment and a primary trigger for CRP production, is highly expressed in various types of tumours [39]. Secondly, tumour cells are capable of producing CRP on their own and might also generate and release cytokines and chemokines like IL-6 and IL-8, which elevate serum CRP levels [40]. Tissue inflammation caused by tumour expansion and invasion results in higher CRP levels [41]. The immune system’s innate and adaptive components may increase CRP levels in response to tumour antigens. Thirdly, patients with elevated CRP show a compromized T-lymphocyte response, which is associated with poor tumour infiltration and negative outcomes [42]. Recent studies have demonstrated that higher CRP levels are inversely related to the infiltration of T-lymphocyte subgroups, potentially contributing to cancer development and progression [37,40]. Therefore, high CRP is associated with poor survival outcomes of melanoma.

Notably, previous studies have demonstrated the prognostic value of CRP flare in melanoma [34]. There are some differences and similarities between CRP flare and CRP baseline levels. First, CRP kinetics consist of the value of pretreatment and posttreatment CRP, whereas CRP baseline is the value of pretreatment. Second, CRP flare-response can be affected by the treatment, whereas CRP baseline levels cannot. Third, they both have prognostic value for patients with melanoma. Moreover, according to a recent study [34], the early kinetics of CRP may reflect a response to ICI in melanoma, resulting in improved OS and recurrence-free survival (RFS)/PFS. CRP flare and response are associated with markedly improved outcomes compared to non-responders in melanoma [34].

Through meta-analysis, CRP has been extensively suggested to play a significant role in forecasting various cancer prognoses [43–47]. According to Feng et al. elevated CRP was significantly related to poor OS, PFS, recurrence-free survival and cancer-specific survival of bladder cancer patients in the meta-analysis including 20 studies [43]. Based on Zhang and colleagues, increased CRP contents in serum showed strong relation to poor OS and PFS of ovarian cancer from the meta-analysis involving 3202 cases [44]. In one latest meta-analysis recruiting 1704 patients, the elevated CRP level was significantly related to inferior OS of glioma [45]. Yang et al. carried out the meta-analysis recruiting 2204 patients, according to their results, higher CRP markedly forecast dismal OS and PFS of cervical cancer cases [46]. Wang et al. conducted the meta-analysis comprising 32 articles, as a result, an increased CRP content before treatment was related to dismal OS and PFS of NSCLC cases undergoing immunotherapy [47]. Therefore, our meta-analysis findings conformed to CRP’s value in forecasting prognosis of additional cancer types.

This study had some limitations. First, among-study heterogeneities were prominent. Therefore, we used random-effects model. Second, there was a small sample size for PFS. Just 5 articles involving 1252 cases were enrolled to analyze PFS. Third, CRP thresholds remained inconsistent among enrolled articles. Due to these limitations, large-scale studies adopting standard CRP cut-off value are still needed to validate our findings in this meta-analysis.

## Conclusion

In summary, this meta-analysis demonstrated that elevated CRP levels significantly predicted poor OS and PFS in patients with melanoma. CRP is the promising biomarker used to forecast melanoma prognosis in clinical practice.

## Supplementary Material

PRISMA_2020_checklist.docx

## Data Availability

The data that support the findings of this study are available from the corresponding author upon reasonable request.
